# The economics of health and climate change: key evidence for decision making

**DOI:** 10.1186/1744-8603-7-18

**Published:** 2011-06-27

**Authors:** Guy Hutton

**Affiliations:** 1 Consultant, Public Health and Environment, World Health Organization, Geneva, Switzerland

## Abstract

**Background:**

In responding to the health challenges of climate change, those responsible for health policies and resource allocations need to know the resource consequences of their decisions. This article examines the availability and strength of economic evidence for policy makers to draw on in making health policy decisions.

**Methods:**

Relevant literature was obtained using a Medline and INTERNET search of key terms and institutions working in health and climate change. Eighteen available economic studies are presented under three categories of economic evidence: health damage cost, health adaptation cost and health economic evaluation.

**Results:**

In economic studies valuing the predicted increased mortality from climate change, the health damages represent an important fraction of overall economic losses. Similarly, when considering broader health protection measures beyond the health sector (e.g. agriculture, water supply) health considerations are central. Global adaptation cost studies carried out so far indicate health sector costs of roughly US$2-5 billion annually (mid-estimates). However, these costs are expected to be an underestimate of the true costs, due to omitted health impacts, omitted economic impacts, and the costs of health actions in other sectors. No published studies compare the costs and benefits of specific health interventions to protect health from climate change.

**Conclusions:**

More economic studies are needed examining the costs and benefits of adaptation measures to inform policy making. There is an urgent need for climate change-specific health economic guidelines to ensure robust methods are used, giving comparable results. Broader advocacy and focused training of decision makers is needed to increase the uptake of economic evidence in decision making. Until further climate change-specific economic studies have been conducted, decision makers should selectively draw on published studies of the costs and benefits of environmental health interventions.

## Background

In responding to the health challenges of climate change, those responsible for international and national health policies and budget allocations need to know the resource consequences of their decisions. These include the size of costs, benefits and financing of policy implementation, the distribution of gains, as well as unintended or negative consequences of health policies. Economic research attempts to answer these questions. However, what economic evidence can policy makers actually draw on in making their decisions regarding climate change? And how accessible is that evidence?

The global evidence base on the economics of climate change is extremely weak, especially in the health sphere. Little is known still on the precise health impacts of climate change at a sub-national level, their economic costs, and the costs and benefits of measures to protect health from climate change. Also, given that climate change is a long-term event, there is currently very limited information on how economic development generally, and health sector development in particular, will protect the health of populations from future climate change, especially in poor but rapidly developing nations. Therefore, having a better understanding of the costs and benefits of health policies and programmes is one concrete way to assist policy makers in making better decisions. The aim of this article is to examine the availability and strength of economic evidence for policy makers to draw on in making health decisions, and to provide recommendations for future evidence generation and dissemination.

## Methods

Relevant literature was obtained using a Medline and INTERNET search of key terms and institutions working in health and climate change. Studies were screened for whether they presented quantitative health economic data on climate change, using title and abstract (where available). From 37 publications initially screened using title and/or abstract, 18 were found to present original data once the full publication was obtained. Other publications presenting compiled evidence and reviewing economic issues were also collected, and where relevant are reported. For simplicity, economic studies can be broadly divided into three categories:

1. Health damage cost studies estimate the societal costs or benefits of the health impacts of different climate change scenarios. 12 studies were identified and are presented below. Scenarios typically compare business-as-usual with global average temperature rise under different greenhouse gas emissions mitigation measures, and the associated health impacts [1,2]. This approach values the health outcomes in monetary units. The primary purpose of these studies is to provide advocacy material to raise attention to the negative consequences of climate change. Data from these studies also feed into other types of economic assessment (see 3. below).

2. Health adaptation cost studies estimate the costs of alternative measures to reduce, or avert altogether, the health impacts of climate change. 3 global studies and 2 country studies were identified and are presented below. Measures can be a combination of preventive and curative interventions taken by the health sector as well as health-affecting measures in other sectors. The primary purpose of these studies is to enable realistic budgeting for the interventions required for adaptation, now and in the future.

3. Health economic evaluation studies essentially compare the costs with the benefits of health adaptation measures, estimating a return on spending in the form of a cost-effectiveness ratio (such as cost per death averted) or a cost-benefit ratio (monetary return per currency unit spent) [3]. When efficiency measures are provided for a range of alternative adaptation measures, policy makers are enabled to select measures using efficiency and other criteria, based on policy goals. 1 study was identified and is presented below.

The economic evidence base is reviewed under these three categories, focusing on the eighteen available studies presenting economic evidence on climate change and health.

## Results

### Health damage costs

Newspaper headlines that announce the number of "billion dollars lost per year due to climate change" can lend political and public support to new adaptation and mitigation policies. However, the general public, and even policy makers, often do not understand what is behind the numbers, such as which impacts are included and excluded, and the robustness of the data sources and valuation methods.

First, not all health damages can be easily monetized. Many health and environmental impacts are intangible or difficult to measure, such as the loss of quality of life or the loss of life itself. Second, some are indirect such as impact on household income and therefore more difficult to attribute precisely to the health impact. Due to these challenges, most studies value only the most direct and quantifiable economic impacts, leading to a systematic under-valuation of the health effects of climate change. Table 1 classifies different damage costs according to their tangibility and directness, based on author's assessment.

**Table 1:** Classification of damage costs^1^

Impact type	More Direct	More Indirect
**More Tangible**	• Health service use • Related health seeking costs (e.g. transport and time)	• Impact on household income or productive time of individuals • Impact on the wider economy through affected labour

**More Intangible**	• Morbidity (health-related quality of life) • Loss of life	• Stress, trauma • Uncertainty

^1 ^Note the classification proposed gives a general indication of which damage costs tend to be 'more' or 'less' tangible and direct, but these can vary between persons and contexts.

Health damage cost estimates are commonly made as part of broader studies on the overall economic costs of climate change. The majority of economic damage cost studies are global and regional in scope. Studies from the 1990s show total damage costs of climate change equivalent to 1% to 3% of GDP, for a commonly modelled (in those days) average global temperature rise of 2.5°C. The global economic value of loss of life due to climate change varies between around US$6 billion and US$88 billion, in 1990 US$ [4-9]. The contribution of the loss of human life to the overall economic losses varied from 6.5% to as high as 50% [6].

Over time, the range of health impacts included has expanded and the current and future deaths and cases have been estimated based on more precise underlying data on both climate impact and the link to health outcomes. Bosello estimates mortality and years lived with disease due to climate change for six diseases [10]. Using a general equilibrium model the study found that the economy-wide impacts of health impacts are greater than simple aggregation of the health costs of different diseases. In the EU, another study found that climate change would produce net annual economic benefits of € 25 billion in the 2020s due to reduced cold deaths exceeding increased heat-related deaths [11]. These net benefits of climate change gradually reduce over time until the 2080s when, for some scenarios, net health impacts turn negative. The damage costs of increased Salmonella cases in the EU due to higher average temperatures has also been estimated, with an annual damage cost of between € 70-139 million until 2040, based on the average medical treatment cost per case of €3,500 [12].

There are several problems with these damage cost studies. One problem is the exclusion of potentially important impacts. For example, most studies value only increased deaths and omit the value of lost productivity and increased health care expenditure. Also, most studies include only a small range of diseases, usually temperature-related and malaria, and omit other climate-sensitive health impacts, such as extreme weather events, waterborne diseases, air pollution and airborne diseases, and other vector-borne diseases such as tick-borne encephalitis or dengue fever.

A second problem is the inherent problem of valuing life. Values are often widely diverging between different studies. Also, global studies value loss of human life based on the GDP of each country, hence giving higher values to richer countries [13]. The unwanted policy consequence of this approach is that adaptation resources would appear to have greater return in richer than in poorer countries.

A third problem is the valuation of impacts far into the future. At larger discount rates, future welfare impacts quickly reduce to a small proportion of their impacts at present values. On the one hand, it is argued that low or zero discount rates for long-term projects accord with the way people think (reflecting the time preference method of setting discount rates) [14]. On the other hand, a low or zero discount rate leads to distorted capital markets, given that most investments are based on the short-term opportunity cost of capital [15]. There is still no consensus on the most appropriate discount rate for measuring climate change impacts.

A second type of damage cost study emerging in the climate change field assesses the damage costs averted of (localised) health co-benefits of mitigation measures. To date, such studies are very few [16,17]. For example, the impact assessment accompanying the European Commission Climate and Energy Package indicated that by reducing greenhouse gas (GHG) emissions by 20% in 2020, sulphur dioxide, nitrogen oxides and PM2.5 emissions would be reduced by 10-15% compared to baseline emissions in 1990, thus reducing health damage costs by between € 12-29 billion [18]. In the EU, China and India, health gains are valued at US$ 145 billion in 2030 from low carbon electricity generation, compared to a business-as-usual scenario [19]. The key message for policy makers of these studies is that carbon reduction measures should not be viewed as only costing money, because they also bring immediate and valuable health benefits. The implication is that taking into account health gains can influence the type of carbon reduction measures selected.

### Health adaptation costs

More recently, attention has been turning from damage cost studies to adaptation cost studies, for which there exist global, regional as well as national studies [20,21]. While there are very few examples so far of health adaptation cost studies, there are ongoing efforts by countries to estimate costs of implementing adaptation strategies in the context of National Adaptation Programmes of Action (NAPA) whose main goal is to identify priorities for adaptation to climate change in diverse sectors. Health adaptation planning methodology has been supported by guidance from the World Health Organization [22], and more recently updated vulnerability, impact and adaptation (V&A) assessment guidelines [23].

Two global health adaptation cost assessments have been conducted as part of multi-sectoral assessments, namely the World Bank study "Economics of Adaptation to Climate Change" in 2010 [24] and the UNFCCC report "Investment and Financial Flows to Address Climate Change" in 2007 [25]. A third global study focuses on health adaptation costs only [26]. All of these studies include diarrheal diseases, malaria and malnutrition. Only the World Bank study explicitly takes into account future economic development, and therefore increased health resilience to climate impacts. The adaptation costs are estimated as the number of additional cases attributed to climate change multiplied by the unit cost of health interventions, consisting of a mixture of preventive and treatment interventions. The results of these studies are compared in Table 2. For the multi-sectoral studies, the sectors in which actions have direct health benefits such as water supply, agriculture, fisheries and extreme weather, are also presented. Table 2 shows the proportion of adaptation costs directly relevant to health protection vary from 14% to 47%, depending on whether high or low cost estimates are used. In the World Bank study, the share of GDP of overall adaptation costs declines from 0.22% in the decade 2010-19 to 0.12% in the decade 2040-49, due to the increased resilience to climate change provided by economic growth.

**Table 2:** Global annual cost of climate change adaptation from the literature, in billion US$

Sector	World Bank^1^ (2005 prices)	UNFCCC^2^ (2007 prices)	Ebi^3^ (2001 prices)
**Period or time point**	**2010-2050**	**2030**	**2030**

Health sector	2.0	3.8 - 4.4	3.3 - 10.7

Water supply	13.7	9.0 - 11.0	-

Agriculture, forestry and fisheries	7.6	14.0	-

Extreme weather	6.7	-	-

**Total health-related**	**30.0**	**26.8 - 29.4**	**3.8 - 4.4**

**Total (all)**	**89.6**^4^	**56.8 - 193.4**^5^	**-**

**% health-related**	**33.4%**	**13.8 - 47.1%**	**-**

'-' not estimated

^1 ^The World Bank study estimates the adaptation costs of two scenarios over four decadal periods from 2010 until 2050. The scenario presented in the table is from the National Centre for Atmospheric Research (NCAR) which is labelled the 'Wettest scenario'. For the other scenario from the Commonwealth Scientific and Industrial Research Organization (CSIRO), labelled the 'Driest scenario', the costs are as follows: human health (US$ 1.6 billion), water supply and flood protection (US$ 19.2 billion), agriculture forestry and fisheries (US$ 7.3 billion), extreme weather events (US$ 6.5 billion).

^2 ^Two scenarios were modelled for the health sector analysis: stabilisation of CO_2_-equivalent greenhouse gases at 750 parts per million by volume (ppmv) by 2210 and 550 ppmv by 2170. The table presents results for 550 ppmv. For the 750 ppmv scenario, the costs vary US$ 4.5 to US$ 5.4 billion. The variation is accounted for mainly by uncertainties in the number of additional malaria cases. In the water sector, the two figures represent SRES B1 (lower cost) and SRES A1 scenarios.

^3 ^Scenarios similar to the UNFCCC study, as the latter used disease figures from Ebi (2008). For the health impacts, Ebi drew on the WHO Global Burden of Disease Study. The table presents results for 550 ppmv. For the 750 ppmv scenario, the costs vary US$ 4.0 to US$ 12.6 billion. Under an unmitigated emissions scenario, costs vary from US$ 5.9 to US$ 18.0 billion.

^4 ^Other sectors are infrastructure and coastal zones. Under the driest scenario these account for US$ 43.1 billion, taking the total global costs to US$ 77.7 billion.

^5 ^Other sectors are infrastructure, coastal zones and natural ecosystems. On infrastructure adaptation costs, there is a wide variation in cost between the estimates based on the Munich Re data (US$ 8 billion) and the Association of British Insurance data (US$ 130 billion).

Health adaptation costs are in the same order of magnitude in the three studies. This is partly explained by the fact that they are based on the same underlying health impact data [27]. However, a high level of agreement should not lead to a mistaken conclusion of accuracy of these estimates, and the three studies suffer the same weaknesses [28]:

• Underlying weaknesses in the health estimates [27]. First, the health impact numbers are highly uncertain due to major uncertainties in various input variables, including: the future emissions scenarios; the future impact of climate change on temperature; the link of temperature to other health-affecting climate variables (e.g. rainfall, storms); and the implications for health. Also, underlying health data, such as current health burdens, are also highly uncertain, especially in developing countries where routine health information systems are generally weak. Second, the studies did not consider the full range of climate-sensitive disease burdens. Heat-related impacts, disaster-related (weather) impacts, and infectious diseases other than malaria and diarrhoea have been omitted. The costs of responding to future food insecurity and malnutrition were only partially considered.

• The unit costs of controlling the health impacts are imprecise, drawing on generalized regional estimates, and limited country-level cost data. Unjustified assumptions are made about the intervention set (preventive and curative services chosen) implemented to reduce the disease burden. The studies include the most quantifiable adaptation costs only, such as service delivery, and largely exclude the cost of implementing new policies and of increasing capacity to meet demand. Only costs that are expected to be financed by public agencies, and not private, were included in the World Bank study.

• The adaptation measures assume perfect foresight and do not take into account the additional costs when hedging a range of outcomes (under different climate scenarios) or of mal-adaptation - where the responses to climate change lead to worse health outcomes due to faulty climate predictions.

• The time horizon of the adaptation cost studies extend to a maximum 40 years into the future. However, health impacts are expected to be considerably greater until, or even after, stabilisation occurs some time in the 22^nd ^or 23^rd ^centuries. Furthermore, under more extreme climate change scenarios involving for example higher sea level rise or widespread desertification, currently planned adaptation will need to be revised to prevent more severe predicted health impacts.

• Uncertainty about the development baseline. Future adaptive capacity, including the adaptation effects of predicted future economic growth (especially in developing countries) is highly uncertain and thus difficult to take into account in the estimates. The World Bank explicitly avoids counting the (costs of the) health impacts that would be averted due to ongoing economic development, which increases the resilience of populations to climate change. However, other studies do not explicitly mention their assumptions on the development baseline.

To date, only few examples exist of cost estimates of health sector adaptation plans in the National Adaptation Programme of Action [20]. One study from Bangladesh estimates an average annual adaptation cost in the health sector, from 2010 to 2050, at US$ 18 million per year [29]. This study assumes the adaptation cost is equal to 20% of per capita health spending, for populations affected by five climate-induced diseases (diarrhoea, skin diseases, malaria, mental disorders and dengue). Under the National Economic, Environment and Development Study for Climate Change (NEEDS) project, whose aim is to estimate financing needs to implement mitigation and adaptation measures, Ghana estimates additional resources of US$ 350 million by 2020 to adapt to climate change in the health sector, plus US$ 7.6 million per year for malaria control [30]. In this publication, no detail was provided on the methodology, data sources and original published materials for these estimates.

### Health economic evaluation

A rational decision maker will ask what return or payback they are getting on expenditure and resources they control. There are several types of decision makers, such as government ministries, district offices, health providers, commercial enterprises and households. Each one will have a different perspective on the impacts of climate change, and the costs and benefits of adapting to climate change. Indeed, economic evaluation should be designed to be usable by a range of decision makers, and hence reflect the viewpoints of the various stakeholders. To date, no published economic evaluation studies have specifically examined the costs and benefits of health adaptation in relation to averting the marginal health risks of climate change [21,31]. An unpublished study from Bangladesh estimates the economic efficiency of a package of health adaptation options targeting diarrheal disease, skin problems, mental disorders, malaria and dengue. The study monetizes the saved private health expenditure and productive time of averted illness. The benefit-cost ratio of intervention measures is estimated to be 4.1, with an annual rate of return of 41% [29].

Other economic studies also indicate economic efficiency, while they fall short of indicating the efficiency of climate change-specific health risks. In one study, the value of health benefits were compared to the costs of heat-health early warning systems in Philadelphia [32]. The study estimated incremental financial costs of the system of US$ 210,000, and the model predicted 117 lives were saved over a 3 year period, with a cost of US$ 1,795 per year of life saved. At a value per saved life of US$ 4 million, the societal value of saved lives was estimated at US$ 468 million. However, no climate-specific attribution factor was made. Another study identified the climate change impact on water supply in South Africa; however, health benefits of adaptation options were not included in the cost-benefit analysis [33].

Hence there are very limited studies available to enable decision makers to understand how to most efficiently address the rising burden from climate-sensitive diseases. Current studies provide very imprecise information on costs and benefits. This field is clearly in its infancy. However, economic evaluation studies face a major constraint in evaluating the cost-benefit or cost-effectiveness of interventions to reduce the specific health risks of climate change, due to the imprecision in isolating these additional health risks from already present health risks. Therefore decision makers should draw on existing economic studies on environmental health interventions that are not specific to climate change. Such studies already exist in the fields of water and sanitation, environmental vector control and air pollution [34], as well as a larger number of economic studies on curative services. On the one hand, the additional climate risk may increase the intervention (adaptation) costs, while on the other hand climate change will lead to increases in potential health benefits of these interventions - hence, the overall efficiency of interventions may not change significantly under climate change.

## Conclusions and recommendations

This paper reviewed the economic evidence base to support adaptation decisions to protect health from climate change, revealing large gaps in economic evidence. The analysis suggests that the existing evidence base is generally of low quality, and given the current global nature of many studies, is of limited relevance for decision makers at national level and below. Until mid-2010, only 23% of the UNFCCC-led National Adaptation Programmes of Action were considered to be comprehensive in their health-vulnerability assessment and 27% (8/30) of these health adaptation interventions were considered to be adequate [35]. 3% of budgeted funds were destined for health. An even larger gap exists in assessments of the value of health benefits in a cost-benefit or cost-effectiveness framework, which ideally would be used for making more efficient decisions on adaptation policies and resource allocations [21].

Although evidence is incomplete, all published evidence suggests significant health damage and adaptation costs, which are an important proportion of overall damage costs for climate change. If this is true, it follows that health should be an important component of adaptation support. Furthermore, the short- to medium-term impacts of climate change on health are mainly expected to be exacerbations of existing effects. In this case, we would expect the existing evidence base on effectiveness of interventions to (roughly) apply, and can conclude that much of the expected increased burden could be avoided through scaling up existing cost-effective interventions. Therefore, until further research assesses the efficiency of intervention specifically in the context of a changing environment, it should be considered acceptable for decision makers to draw on existing economic evidence that is non-climate specific. One area of focus should be on the implementation of policies which have a beneficial health impact even with inaccurate predictions of the health impacts of climate change (often termed 'no regrets' policies), which in the short-term can potentially avert significant health impacts, part of which are attributed to climate change.

Three major developments specific to the economics evidence base could inform policy making. First, more climate change-specific health economic guidelines and tools. Second, further review of the existing environmental health economic evidence base to assess relevance for climate change-specific interventions. Third, measures for improved dissemination and communication of economic results within the health sector as well as mainstreamed into all relevant sectors. These three proposed future developments in the economics evidence base are elaborated below.

Improved guidelines and tools: Many of the uncertainties identified in this study can be addressed through more focused health studies at a higher level of resolution at the national level. These studies would also have the advantage of being able to directly inform national and sub-national governments, forming part of the national adaptation strategies and associated fund raising activities. To be robust and standardized, guidelines and tools are needed which should describe the detailed research methodology, outlining clearly the analytical choices, and providing concrete guidance on which methods and values to use in valuing benefits under climate change [36,37]. They should specifically target government departments wishing to make a cost or economic assessment of health adaptation plans. Software-based tools aid the researcher to enter the inputs and generate the outputs in a standardized way. When conducting economic evaluation, analysts should conduct cost-effectiveness and cost-benefit analysis together, to be of wider appeal not only to health ministries (who tend to use CEA) but also other ministries (who are more interested in CBA, especially if it includes benefits relevant to their mission). To increase social efficiency, a wide range of interventions should be evaluated, even if they are under the responsibility of different government departments. For example, to reduce diarrheal disease burden, the economic performance of curative care should be compared with preventive interventions implemented by the health sector such as rotavirus or cholera vaccinations, as well as with preventive interventions under the charge of other ministries such as water resource protection. Multidisciplinary research on climate-health links, adaptation and mitigation measures should include health economic analysis [38]. Given the substantial health gains that can be made through actions in other sectors, it is crucial to adopt a broader multi-sectoral perspective in the cost analysis. Furthermore, health economic guidelines should link climate change adaptation and mitigation from a health policy perspective.

Review of existing economic evidence base on climate change-sensitive health burdens: to date, environmental health economic studies do not specifically incorporate climate change considerations. Therefore, a review of studies should be carried out that provides a climate change angle - for example, assessing how costs and benefits would be altered under climate change. Based on determinants of costs and efficiency - such as underlying disease risk, climate change and variability, relative prices of goods and services, and existing policies and interventions - it needs to be described clearly how evidence from one context can be transferred or extrapolated to other contexts.

Improved dissemination and communication of economic results: to encourage greater use of (economic) evidence in decision making, economic data should be presented in a manner that is easily understood by policy makers. Aside from providing short and digestible summaries of research results to decision makers [39], other improvements are needed in the presentation and use of economic evidence. For example, multi-criteria analysis - which is used either explicitly or implicitly in most decisions - can be expanded to include more economic variables. Opportunities for health gains should be made more explicit to decision makers, with supporting evidence, such as 'no regrets' policies or interventions that have ancillary (co-)benefits. Decision makers in other sectors such as energy, transport, housing, infrastructure, drinking water, agriculture and emergency services also need to be shown how their interventions can be fine-tuned to have greater positive impact on health. Furthermore, using the evidence base more effectively in health policy related to climate change may have positive spill-over effects on the entire health sector, hence bringing greater benefits than just averting the disease burden attributed to climate change.

Future economic studies on climate change and health should not be implemented in isolation from other initiatives. Most importantly, this includes having a strong link with national adaptation activities to promote rational decision making using an improved health economic evidence base. These activities include NAPAs and other capacity-building projects such as UNFCCC's NEEDS project; UNDP's "Capacity Development for Policy Makers to Address Climate Change" whose aim is to promote multi-stakeholder dialogue and conduct assessments in long-term investment and financial flows; and a global project implemented at regional level "Review of the Economics of Climate Change" (RECC) whose aim is to contribute to the regional debate on the costs and benefits of climate change adaptation and mitigation, including advocacy and support to governments and the private sector.

## Competing interests

The author declares that they have no competing interests.
